# Thyroid cartilage resection surgery for metastases of clear cell renal cell carcinoma: case report and literature review

**DOI:** 10.31744/einstein_journal/2026RC2002

**Published:** 2026-02-02

**Authors:** Lorenzo Zanardo Lisboa, Gustavo Barreto da Cunha, Maria Luísa Pedalino Pinheiro, Leonardo Haddad, Fabio Pupo Ceccon

**Affiliations:** 1 Hospital Israelita Albert Einstein Faculdade Israelita de Ciências da Saúde Albert Einstein São Paulo SP Brazil Faculdade Israelita de Ciências da Saúde Albert Einstein, Hospital Israelita Albert Einstein, São Paulo, SP, Brazil.; 2 Universidade Federal de São Paulo São Paulo SP Brazil Universidade Federal de São Paulo, São Paulo, SP, Brazil.; 3 Hospital Israelita Albert Einstein São Paulo SP Brazil Hospital Israelita Albert Einstein, São Paulo, SP, Brazil.

**Keywords:** Thyroid cartilage, Neoplasm metastasis, Carcinoma, renal cell, Larynx, Laryngectomy

## Abstract

Laryngeal cartilage is an exceptionally rare site of metastasis, with fewer than 63 cases reported in the literature; prostate, kidney, breast, and lung cancers are the most common primary tumors. In addition to this extreme rarity, most published reports are limited to descriptions of the lesion and radiologic findings, without detailing management strategies or follow-up outcomes. The limited number of studies exploring surgical intervention have primarily described superficial resections or conventional laryngectomies. In this context, this case report describes thyroid cartilage metastasis of clear cell renal cell carcinoma in an asymptomatic patient, treated with an innovative surgical approach based on isolated thyroid cartilage resection, with a nine-year follow-up.

## INTRODUCTION

Laryngeal metastatic neoplasms are uncommon, accounting for only 0.09%–0.4% of all laryngeal tumors.^(1)^ The first fully described case of distant metastasis to the larynx, reported in 1924, originated from renal adenocarcinoma.^(2)^ Since then, various other cases of secondary laryngeal tumors have been reported, with melanoma being the most frequent histological type, followed by renal cell carcinoma.^(3)^

Although laryngeal metastases are rare, secondary involvement of laryngeal cartilaginous tissue is even more uncommon, with only 63 cases reported in the literature. Comprehensive literature reviews have demonstrated that primary malignancies metastasizing to the thyroid and cricoid cartilage most commonly arise from the prostate, kidneys, breasts, and lungs.

Most published case reports on this topic are limited to descriptions of the lesion and the radiologic findings, with 76% lacking a description of patient treatment or follow-up. The remaining studies vary in their therapeutic approaches, reporting no directed treatment (11%), local radiotherapy (6%), or surgical procedures (6%). Among the few surgically treated cases, two total laryngectomies, one partial laryngectomy, and one isolated tumor resection have been performed, using what are presumed to be conventional techniques.^(4–7)^

This case report describes a thyroid cartilage metastasis of clear cell renal cell carcinoma treated with an innovative surgical approach based on isolated thyroid cartilage resection, with follow-up extending from 2015.

## CASE REPORT

**Figure 1:**
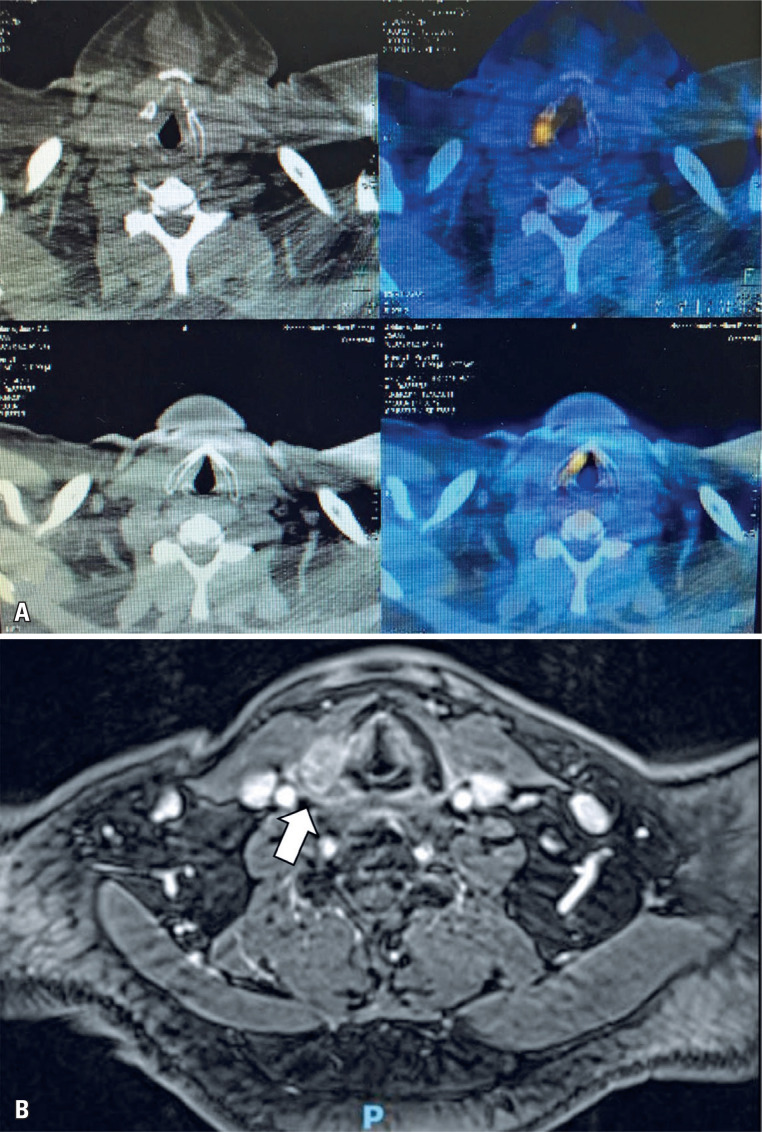
A) PET-CT revealed increased fluorodeoxyglucose (FDG) uptake in a hypermetabolic lesion involving the right thyroid cartilage ala; B) MRI showed an infiltrative lesion of the right thyroid cartilage, without invasion of other structures (arrow)

A 54-year-old man with a history of clear cell renal cell carcinoma, previously treated with partial nephrectomy, was admitted to the Otorhinolaryngology and Head and Neck Surgery Department one year after initial neoplasm treatment following identification of a hypermetabolic lesion with a lytic appearance in the right thyroid cartilage ala on positron emission tomography (PET) (Figure 1A). Positron emission tomography-computed tomography (PET-CT) also revealed a bone metastasis in the humerus, which was surgically resected. On physical examination, the patient was completely asymptomatic, with no voice alterations, dysphagia, or cervical complaints, and denied smoking or other toxic exposures. Airway and neck examinations were normal, as was videolaryngoscopic evaluation.

Magnetic resonance imaging (MRI) showed an infiltrative lesion involving the right thyroid cartilage, without invasion of other structures (Figure 1B), and fine-needle aspiration was subsequently performed. Histopathological results were consistent with the thyroid cartilage metastasis of clear cell renal cell carcinoma. Owing to the rarity of this case, surgery was proposed to achieve tumor resection and provide better survival outcomes.

**Figure 2:**
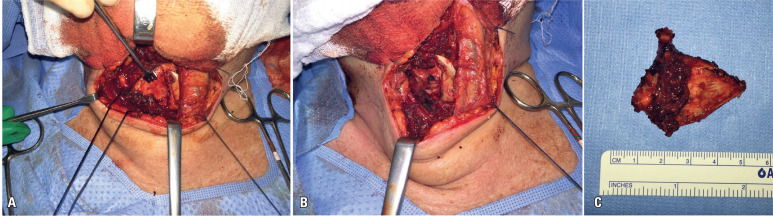
Intraoperative images showing extraction of the right hemi-thyroid cartilage (A), preservation of laryngeal integrity through conservation of the internal perichondrium (B), and the excised right hemi-cartilage containing the metastatic lesion (C)

The procedure was performed under general anesthesia with orotracheal intubation and consisted of a transverse cervical incision, right hemicartilage resection, and complete excision of the metastatic lesion, while preserving the integrity of the internal perichondrium and maintaining an unaltered laryngeal lumen (Figure 2). After achieving hemostasis and layered reconstruction, a preventive tracheostomy was performed.

The patient recovered completely, and the tracheostomy was subsequently closed. After nine years of follow-up, no disease recurrence or functional impairment was observed.

Written informed consent was obtained for publication of this case, and the accompanying images, and the study was approved by the Research Ethics Committee of *Hospital Israelita Albert Einstein* under the CAAE: 88872325.3.0000.0071; 7.615.519.

## DISCUSSION

Thyroid cartilage metastases from renal cell carcinoma are uncommon. Among the 63 reported cases of laryngeal cartilage metastasis in the literature, 29 originated from prostate cancer, eight from breast tumors, eight from renal neoplasms, and other sites and histologies, including the lungs (six), melanoma (three), and leukemia (three).

The rarity of secondary involvement of cartilaginous tissue is attributed to its limited vascular supply and sparse lymphatic drainage. However, the prevailing hypothesis proposes that focal ossification permits hematogenous metastatic seeding within small medullary spaces formed during this process.^(8)^

Clinically, it can generate various symptoms, including hoarseness, dyspnea, and stridor, or may remain completely asymptomatic (60.5% of the cases fully described), as in our case.^(3)^ Nonetheless, this subtype of laryngeal metastasis is most often an incidental finding on laryngoscopic and radiologic examinations (such as PET-CT), and is considered a marker of poor prognosis, generally indicating synchronous metastases to other organs, including the bones, lungs, and liver.^(9)^

Despite the poor prognosis and clinical heterogeneity of these patients, limited information is available regarding management strategies, as only 15 reports have described any form of treatment or patient follow-up. Among these, eight attempts were made to directly treat cartilaginous metastases (four with radiotherapy and four with surgical intervention).

The surgical approaches described to date include two total laryngectomies, one partial laryngectomy, and one isolated tumor resection. In the present case, isolated resection of the hemi-cartilage with preservation of the internal perichondrium was successfully achieved. This approach represents a valuable surgical option that allows maximal functional preservation and reduced surgical morbidity, while achieving complete disease remission. Furthermore, despite the limited experience reported in the literature, laryngeal cartilage metastasis appears to impose a high risk of subglottic stenosis at the time of diagnosis (three of 15 cases with reported follow-up), which may favor a surgical treatment strategy.

## CONCLUSION

Metastasis to the laryngeal cartilage is rare, and both treatment approaches and patient follow-up data remain extremely scarce in the literature. Given the risk of subglottic stenosis and the generally poor prognosis, isolated thyroid cartilage excision with preservation of the internal perichondrium should be considered a feasible and innovative surgical treatment option.

## Data Availability

The underlying content is contained within the manuscript.
